# School Climate and Sleep Duration Among Adolescents at the Intersection of Multiple Social Positions

**DOI:** 10.1002/jad.70105

**Published:** 2026-01-15

**Authors:** André Gonzales Real, Brian T. Gillis, Marla E. Eisenberg, G. Nic Rider, Benjamin Parchem, Samantha E. Lawrence, Stephen T. Russell

**Affiliations:** ^1^ Department of Psychiatry and Behavioural Neurosciences McMaster University Hamilton Ontario Canada; ^2^ Department of Human Development and Family Science Auburn University Auburn Alabama USA; ^3^ Division of General Pediatrics and Adolescent Health, Department of Pediatrics University of Minnesota Minneapolis Minnesota USA; ^4^ Department of Family Medicine and Community Health Institute for Sexual and Gender Health University of Minnesota Medical School Minneapolis Minnesota USA; ^5^ Potocsnak Family Division of Adolescent and Young Adult Medicine, Pritzker Department of Psychiatry and Behavioral Health Ann & Robert H. Lurie Children's Hospital of Chicago Chicago Illinois USA; ^6^ Department of Psychiatry and Behavioral Sciences Northwestern University Feinberg School of Medicine Chicago Illinois USA; ^7^ School of Social Work University of Connecticut Hartford Connecticut USA; ^8^ School of Social and Family Dynamics Arizona State University Tempe Arizona USA

**Keywords:** adolescents, intersectionality, school climate, sleep

## Abstract

**Introduction:**

Recent studies have indicated that sleep is fundamental for adolescents' physical and mental health. Although it is known that context influences sleep, the impact of school climate on sleep duration remains understudied.

**Methods:**

Using a large, diverse, population‐based sample of adolescents attending California high schools (*N* = 277,954; data collection: 2018–2019) and applying two statistical methods suggested for quantitative research using an intersectionality approach (linear regressions with interaction terms and Exhaustive Chi‐square Automatic Interaction Detection [ECHAID]), this study examined associations between school climate and sleep duration among adolescents at the intersection of multiple social positions.

**Results:**

Similar proportions of participants were assigned male and female at birth. The sample was racially and ethnically diverse (54.1% Latina/x/o). The large majority of participants were straight (85.4%) and cisgender (97.7%). On average, participants slept 6.75 h/night. Positive school climate was associated with longer and adequate sleep duration; however, this association varied across social positions, such that the effects of school climate on sleep duration were attenuated among adolescents who held some minoritized social positions. ECHAID results indicated that those reporting the lowest averages of sleep duration not only perceived school climate as negative but also held multiple minoritized identities. In contrast, those who perceive their school climate as positive are overrepresented among those who reported the highest averages of sleep duration.

**Conclusion:**

Findings underscore the impact that schools have on adolescents' sleep health. Our study indicates that adolescents with multiple minoritized social positions face additional challenges impacting their sleep. Future interventions should focus on strategies to improve school climates, given that they would benefit a large number of students.

About three‐quarters of adolescents attending high schools in the United States are not getting the recommended 8 h of sleep daily, according to the National Sleep Foundation (Hirshkowitz et al. 2015; Kann et al. 2018). Worldwide, lack of sleep among adolescents has been a growing public health concern (Gariepy et al. 2020; Gradisar et al. 2011) because it is associated with multiple physical and mental health problems including obesity, poor emotion regulation, and suicidality (Clark et al. 2023; Matricciani et al. 2019). Scholars have indicated that contextual factors influence how much sleep adolescents obtain (Mayne et al. 2021; Troxel et al. 2017), with lack of school safety associated with inadequate sleep (Meldrum et al. 2018). A growing literature on disparities for minoritized adolescents has shown that youth with multiple minoritized social positions or identities—their sex, racial or ethnic identity, gender modality, and sexual orientation—report comparatively more negative school climates (Gower et al. 2023), as well as higher rates of inadequate sleep, than those in more advantaged positions (Marczyk Organek et al. 2015; Real et al. 2024; Saelee et al. 2023). An intersectionality framework offers insights into these disparities by considering that the intersection of social positions carries unique experiences within overlapping systems of privilege and oppression (Bowleg 2012; Crenshaw 1991). Such systems of social power may determine which schools have access to resources and/or which students can benefit from these resources at school; these factors, in turn, influence perceptions of school climate. Notably, these systems of social power may also expose adolescents holding minoritized social positions (and the intersection of these social positions) to experiences of stigma (e.g., sexism, racism, transphobia, homophobia), which may amplify experiences of chronic stress that can affect sleep. The current study brings together these separate research findings by exploring the role of school climate in understanding disparities in sleep duration among adolescents with multiple intersecting minoritized social positions.

## 1 Disparities in Sleep Duration and Intersectionality

Disparities in sleep duration between adolescents from minoritized social positions have been documented: sexual and gender minoritized adolescents, as well as those from lower socioeconomic status and from minoritized racial and ethnic groups, sleep less on average than those from more advantaged groups (Clark et al. 2023; Hawkins and Takeuchi 2016; Kann et al. 2018; Levenson et al. 2021; Real et al. 2025; Saelee et al. 2023). This pattern is consistent with health disparities for minoritized groups that occur as a function of chronic exposure to rejection and discrimination (e.g., discrimination based on gender, racial and ethnic identity, and sexual orientation; Brooks 1981; Meyer 2003). Only a few studies, however, examine how the intersection of social positions may be associated with adolescents' sleep (Dai et al. 2020; Marczyk Organek et al. 2015; Real et al. 2024, 2025; Saelee et al. 2023); those studies indicate that youth who hold intersecting minoritized social positions experience shorter sleep compared to those holding more privileged social positions. Importantly, the intersection of social positions may represent the unique experiences of individuals in the interlocking societal systems of privilege and oppression which may shape their health and behaviors (Bowleg 2012). For example, adolescents holding racial, ethnic, and sexual minoritized social positions may be exposed to bias‐based discrimination both due to their racial, ethnic, and sexual identities, which in turn have been associated with higher levels and rates of emotional distress (Eisenberg et al. 2024; Mallory and Russell 2021).

## 2 School Climate and Sleep Health

Research on school climate has received substantial attention in recent years (Aldridge and McChesney 2018; Ioverno and Russell 2021, 2022; Long et al. 2021). School climate is a complex construct characterized by the quality of the school, which encompasses, but is not limited to, perceptions of safety, engagement with activities, and relationship with peers and adults at school (Thapa et al. 2013). Scholars have consistently documented that school climate and its dimensions are associated with adolescents' well‐being (see Aldridge and McChesney 2018; Mori et al. 2021 for systematic reviews). Positive school climate has been linked to better mental health (Ford et al. 2021; Leurent et al. 2021; Long et al. 2021; Suldo et al. 2012; Wang et al. 2020), including lower rates of depressive symptoms and suicidality among adolescents (Moore et al. 2018).

Although all adolescents have the right to experience their school climate as positive, this is not the case for many minoritized adolescents. For example, transgender and gender diverse adolescents are more likely to perceive their school climate as negative and avoid school because of safety concerns (Day et al. 2018), and sexual minoritized adolescents perceive their school climate as more hostile than their heterosexual peers (Ioverno and Russell 2022). Other work has shown that within the same schools, Black students report different perceptions of school climate compared to White students, including less supportive relationships (Bottiani et al. 2016; Konold et al. 2017). However, the association between school climate and adolescent well‐being varies by one's access to systems of power and one's experiences of oppression related to their social positions. For example, Dessel et al. (2017) found that gender and sexual minoritized adolescents experienced fewer benefits of positive school climates on their self‐esteem than did their cisgender and heterosexual peers.

Despite the importance of sleep for adolescents' physical and mental health (Clark et al. 2023; Matricciani et al. 2019), it is surprising that only one known study has examined how school climate may be associated with sleep outcomes (Meldrum et al. 2018). That study found that lower perceived school safety is linked to insufficient sleep among adolescents (Meldrum et al. 2018). School safety, however, is just one aspect of school climate. Further, it is unclear whether this association differs by adolescents' social positions, as has been shown for associations with well‐being outcomes (Aldridge and McChesney 2018; Dessel et al. 2017; Nijs et al. 2014). Given that adolescents holding multiple minoritized social positions perceive their school climates as more hostile (Gower et al. 2023; Ioverno and Russell 2022), school climate perceptions possibly vary by intersecting social positions and contribute to sleep disparities.

## 3 The Current Study

Guiding this present study, we applied an intersectionality approach to understand the ways societal systems of oppression and privilege are reflected in intersecting social positions that shape adolescents' well‐being (Bowleg 2012; Crenshaw 1991). Recent scholarship identifies the limitations and possibilities for quantitative analytic approaches to investigate the complex interplay of multiple social positions (Bauer et al. 2021). In one approach, categorical measures of social positions are used in traditional linear regression to identify independent main effects of each social position, as well as statistical interactions among these measures of social positions. Yet, such models are limited for understanding complex patterns of social positions. When considering more than a few social positions, the possible combination of interactions (e.g., measures of social position could include sex assigned at birth, racial or ethnic identity, gender modality, and sexual orientation) becomes infeasible for regression analyses and interpretation (e.g., in three‐ or four‐way interactions). Scholars have proposed employing decision‐tree methods in intersectional research as an alternative to statistical interactions in regression frameworks (Bauer et al. 2021). Decision‐tree methods estimate every interaction between multiple categorical social positions, maximizing the identification of groups with similar rates or averages of the outcome of interest. Such an approach addresses limitations when the possible combinations of interactions become unwieldy in traditional regression approaches.

For this study, we use these two complementary methods. First, we investigated whether school climate perceptions are associated with sleep duration among a large and diverse statewide adolescent sample using a predictive framework with linear regressions. We applied interaction terms between school climate and each social position to test whether the associations between school climate and sleep duration varied across social positions. Then, using a decision‐tree approach, we examined how and whether perceptions of school climate intersect with multiple social positions in association with adolescents' sleep duration.

## 4 Methods

We utilized data from the 2018–2019 California Healthy Kids Survey (CHKS). CHKS is a school‐based statewide survey, administered biennially to adolescents in 7th, 9th, and 11th grade classrooms from California public schools (a small proportion of students in other grades also completed the survey because they were attending 7th, 9th, or 11th grade classes). Participants have the option to complete the survey on paper or online, and in English or Spanish. The California Department of Education requires that school districts conduct a core survey module; some schools administer supplemental survey modules. More information about the CHKS can be found elsewhere (California Department of Education Safe and Healthy Kids Program Office & WestEd Health and Human Development Department 2019).

Given that sleep problems exacerbate in high school years (Saelee et al. 2023), this study focused on students from 9th to 12th grades. Participation rates ranged between 56% and 70% depending on grade level. Around 2% of surveys were dropped from the analyses because of validity concerns (i.e., a pattern of highly improbable responses or acknowledgment of untruthful responses). Likewise, about 5% of surveys were excluded because schools did not ask participants about their sex assigned at birth (a covariate in the analysis, as described below). Our final analytic sample included 277,954 adolescents attending high school who responded to the question assessing sleep duration.

### 4.1 Measures

#### 4.1.1 Social Positions

We included grade (as a proxy for age) and four social positions for the current study: sex assigned at birth, racial/ethnic identity, sexual orientation, and gender modality. Given the sample design (i.e., the survey was administered in 9th and 11th grade classrooms, which included small numbers of 10th and 12th grade students), and more similar averages of sleep duration among those in the 9th and 10th (9th: 6.99 h; 10th: 6.79 h) and among those in the 11th and 12th grades (11th: 6.50 h; 12th: 6.50 h), we grouped participants in 9th and 10th grades, and 11th and 12th grades. Sex assigned at birth was assessed with the question: “What is your sex?” (Male, Female). A measure for racial/ethnic identity was created in two steps. First, adolescents reported whether they had a Hispanic or Latino ethnicity (Yes, No). In a second question, participants reported their racial identity (American Indian or Alaska Native, Asian, Native Hawaiian or Pacific Islander, Black or African American, White, Multiracial). Hispanic/Latino participants were coded as Hispanic/Latina/x/o (María del río‐González 2021), regardless of their racial identity; for those who were non‐Hispanic/Latina/x/o (NL), participants' selected racial identity was used. Participants also reported their sexual orientation (straight, gay or lesbian, bisexual, I am not sure yet, something else, decline to respond) and their gender modality (cisgender, transgender, not sure, decline to respond). Participants who were not sure of their sexual orientation or gender modality were coded as questioning, and those who declined to respond were coded as missing (but retained for analysis, as described below).

#### 4.1.2 Sleep Duration

Participants were asked: “On an average school night, how many hours of sleep do you get?” In whole hours, response options ranged from 4 h or less (=4) to 10 or more hours (=10).

#### 4.1.3 School Climate

Following previous work utilizing multiple measures for this complex construct (Hanson 2012; Ioverno and Russell 2021, 2022), 15 items measured school climate by assessing four domains: school safety (two items; e.g., “I feel safe in my school”), caring relationships (six items; e.g., “In my school, there is a teacher who believes I will be a success”), school connectedness (four items; e.g., “I feel like I am part of this school”), and meaningful participation (three items; e.g., “I do interesting activities at school”). This school climate scale has been previously validated (Hanson 2012), including for sexual and gender minoritized samples (Ioverno and Russell 2022), and had high internal reliability in this sample (α = 0.90). Items had different response options depending on the domain. The two school safety items were assessed on 5‐point Likert scales (for one, response options ranged from strongly disagree to strongly agree, and the other response options ranged from very unsafe to very safe). Caring relationships and meaningful participation items were measured on a 4‐point scale ranging from not at all true to very true. School connectedness items were measured on a 5‐point Likert scale ranging from strongly disagree to strongly agree. Given different scales, we standardized each item using *z*‐scores, and a summary scale of all 15 items was generated by calculating the mean of each standardized score (ranging from –2.89 to 1.78) (Ioverno and Russell 2022). Higher scores indicate perceptions of a better school climate. Of note, 79 participants were missing all school climate items and, therefore, were excluded from the present analyses.

##### 4.1.3.1 Data Analysis

Data were analyzed using SPSS version 28 and Stata 18.0. First, descriptive statistics and ANOVAs compared the average sleep duration and school climate scores among adolescents based on their grade and each social position separately (i.e., race/ethnicity, sexual orientation, gender modality, and sex assigned at birth). Post‐hoc pairwise comparisons with Bonferroni corrections for multiple comparisons were also conducted.

Given the nested structure of the data, with adolescents nested within schools, an unconditional hierarchical linear model was conducted to determine whether between‐school differences were substantial and hierarchical linear models would be required for the main analysis. The intraclass correlation (ICC) indicated that only 2.3% (ICC = 0.023) of the variance in sleep duration was due to between‐school factors. Because this finding indicates that only a small amount of sleep duration variance is attributed to adolescents' school, the main analyses were conducted using linear regressions. We then conducted multivariable linear regression analyses, adjusted for adolescents' social positions, to test associations between school climate perceptions and sleep duration (α was set at *p* < 0.001 due to the very large sample). For reference groups for categorical measures in linear regression analyses, non‐missing categories with highest average sleep duration based on the ANOVA analyses were used. A set of linear regressions was conducted to examine whether grade and social positions moderated the relationship between school climate perceptions and sleep duration among adolescents: we tested interaction terms between school climate scores and grade, and each social position separately. Omnibus tests (multivariate Wald tests) were conducted to indicate whether the inclusion of the interaction improved the model fit. Given that all models that included interaction terms produced statistically significant results and improved the model (Wald tests *p* < 0.001), we tested stratified models to illuminate the effects of school climate on sleep duration for each category. In addition, in order to examine the effects of each domain of school climate, a similar set of stratified models was conducted to explore whether some domains are more or less beneficial for sleep duration depending on adolescents' social position (shown in Supporting Materials).

Next, we conducted exhaustive chi‐square automatic interaction detection (ECHAID) analysis to examine the average sleep duration among adolescents at the intersections of the multiple social positions. ECHAID utilizes a decision‐tree approach that cycles through all possible interactions between predictors (i.e., social positions, school climate), using successive chi‐square tests. ECHAID generates a tree by creating splits (beginning with the predictor with the smallest *p*‐value) when predictors have significant differences in the average of sleep duration. The splitting process continues until it identifies a “terminal node” by not finding significant differences between categories (Bonferroni‐corrected *p*‐value < 0.05) or when the model constraints are met (minimum terminal node sample size of 100 to avoid overfitting; depth of 5). To ensure that results are robust, we utilized 10‐fold cross‐validation. Importantly, in ECHAID, missing values are retained in the analysis as a “missing” category. These procedures follow previous studies using ECHAID (e.g., Eisenberg et al. 2022). For the present study, we included grade, four social positions, and school climate perceptions in the model to examine how averages of sleep duration vary for participants at multiple and distinct intersections of social positions and perceptions of school climates. Given that ECHAID produces more interpretable results with categorical variables, for this analysis, school climate *z*‐scores were coded based on percentile, indicating negative (<25%), average (25%–75%), and positive (>75%) school climate perceptions (Table S1). We present the 10 intersectional groups with the highest and the lowest averages of sleep duration to illustrate observable patterns of disparity.

## 5 Results

Sample descriptive statistics are shown in Table 1. The sample was roughly evenly divided for 9th and 10th graders (53.3%) versus 11th and 12th graders (46.7%), and similar proportions of participants were assigned male and female at birth. The sample was also racially and ethnically diverse, with 54.1% of participants identifying as Latina/x/o. The large majority of participants were straight (85.4%) and cisgender (97.7%). Overall, the full sample reported an average of 6.75 h of sleep. When looking at average sleep duration across social positions, those who were in 9th or 10th grade, assigned male at birth, identified as NL White (not significantly different than Native Americans), straight, and cisgender reported the highest averages for sleep duration (Table 1). For example, straight adolescents had an average sleep duration of 6.81 h, which differed significantly from 6.36 h among gay/lesbian adolescents (denoted by the “u” superscript), 6.27 h among bisexual adolescents (denoted by the “v”), 6.55 h among questioning adolescents (denoted by “x”), 6.28 h among adolescents who reported “something else” for sexual orientation, and 6.76 h among adolescents who did not share their sexual orientation. Better school climates were perceived among those who were in 9th or 10th grades, assigned male at birth, identified as NL White, straight, and cisgender. Table S1 (Supporting Materials) shows the raw averages of school climate domains for each social position. Table S2 presents the distribution of participants across negative, average, and positive school climates; a larger proportion of adolescents holding racial and ethnic, gender, and sexual minoritized social positions perceived their school climate as negative.

**TABLE 1:** Sample sociodemographic characteristics, and average of sleep duration and school climate across participants.

Characteristic	*N* (%)	Average of sleep duration (SD)	*F*	Average of school climate (SD)	*F*
**Grade**					
9th or 10th	148,011 (53.3)	6.99 (1.44)^a^	7245.6*	−0.00 (0.64)	2.4*
11th or 12th	129,943 (46.7)	6.50 (1.40)^a^		0.00 (0.65)	
**Sex assigned at birth**					
Male	134,850 (49.8)	6.86 (1.44)^b,c^		0.01 (0.65)^a.b^	
Female	136,116 (50.2)	6.63 (1.41)^b,d^	863.2*	−0.01 (0.62)^a,c^	79.3*
Missing	6988 (2.5)	6.69 (1.50)^c,d^		−0.07 (0.69)^b,c^	
**Racial and ethnic identity**					
NL Native American	2024 (0.7)	6.81 (1.58)^e,f,g^		−0.03 (0.71)^d,e^	
NL Asian/Pacific Islander	33,544 (12.2)	6.53 (1.38)^e,h,i,j,k,l^		0.06 (0.61)^d,f,g,h,i,j^	
NL Black	8698 (3.2)	6.64 (1.54)^f,h,m,n,o^	184.9*	−0.07 (0.67)^f,k,l^	614.2*
Latina/x/o	148,850 (54.1)	6.78 (1.44)^i,m,p,q,r^		−0.05 (0.63)^g,m,n^	
NL White	59,600 (21.7)	6.82 (1.38)^j,n,p,s^		0.11 (0.65)^e,h,k,m,o,p^	
NL Multiracial	22,616 (8.2)	6.68 (1.47)^g,k,q,s,t^		−0.02 (0.64)^i,l,n,o,q^	
Missing	2622 (9.4)	6.88 (1.52)^e,l,o,r,t^		−0.04 (0.69)^j,p,q^	
**Sexual orientation**					
Straight	227,841 (85.4)	6.81 (1.40)^u,v,x,y,z^		0.03 (0.62)^r,s,t,u,v^	
Gay or Lesbian	5363 (2.0)	6.36 (1.53)^u,w,aa,ab^		−0.13 (0.68)^r,w,x^	
Bisexual	18,498 (6.9)	6.27 (1.47)^v,w,ac,ad^	724.7*	−0.16 (0.63)^s,y,z,aa^	660.9*
Questioning	10,727 (4.0)	6.55 (1.47)^x,aa,ac,ae,af^		−0.09 (0.63)^t,w,y,ab^	
Something else	4298 (1.6)	6.28 (1.64)^y,ae,ag^		−0.20 (0.69)^u,x,z,ab,ac^	
Missing	11,227 (4.0)	6.76 (1.63)^z,ab,ad,af,ag^		−0.11 (0.71)^v,aa,ac^	
**Gender modality**					
Cisgender	261,660 (97.7)	6.76 (1.41)^ah,ai,aj^		0.02 (0.62)^ad,ae,af^	
Transgender	2629 (1.0)	6.14 (1.73)^ah,ak,al^	225.9*	−0.35 (0.73)^ad,ag,ah^	826.9*
Questioning	3563 (1.3)	6.42 (1.69)^ai,ak,am^		−0.25 (0.68)^ae,ag,ai^	
Missing	10,102 (3.6)	6.72 (1.71)^aj,al,am^		−0.13 (0.73)^af,ah,ai,^	

*Note: N* = 277,954. Percent of missing calculated based on full N. All valid response options are shown as the valid percent (excludes missing values).

Abbreviation: NL = Non‐Latina/x/o.

* *p* < 0.001. Columns with the same letters significantly differ between groups.

Results from the linear regression analysis indicate that school climate perceptions have a significant positive association with sleep duration for adolescents (*b* = 0.42, *p* < 0.001). Given that a coefficient equivalent of one would indicate a difference of approximately 1 h of sleep, our results show that each standard deviation on the school climate scale was associated with approximately 25 min of sleep, on average. The positive association of perceptions of school climate with sleep duration varied by grade and each of the four social positions examined here, as evidenced by statistically significant omnibus tests of interaction (*p* < 0.001). For example (as shown in Table 2), the positive association between school climate perceptions and sleep duration was weaker for participants who were in 11th or 12th grades (11th or 12th: *b* = 0.33 [99% CI 0.32–0.35] vs. 9th or 10th: *b* = 0.49 [99% CI 0.48–0.51]), assigned female at birth (females: *b* = 0.39 [99% CI 0.38–0.41] vs. males: *b* = 0.44 [99% CI 0.43–0.46]), were racial/ethnic minorities (except for NL Native American and NL Multiracial; NL Asian/Pacific Islander: *b* = 0.33 [99% CI 0.30–0.36], NL Black: *b* = 0.35 [99% CI 0.29–0.42], Latina/x/o: *b* = 0.39 [99% CI 0.38 – 0.41] vs. NL White: *b* = 0.45 [99% CI 0.42–0.47]), and those who identified as gay or lesbian (gay or lesbian: *b* = 0.32 [99% CI 0.25–0.40] vs. straight: *b* = 0.41 [99% CI 0.40–0.43]).

**TABLE 2:** Multiple linear regressions examining the effects of social positions and school climate perceptions on sleep duration.

	Main effects for school climate in models stratified by social positions*	
	*b*	*99% CI*
**Grade**		
9th or 10th Grade	0.49^a^	[0.48, 0.51]
11th or 12th Grade	0.33^a^	[0.32, 0.35]
**Sex Assigned at Birth**		
Male	0.44^b^	[0.43, 0.46]
Female	0.39^b^	[0.38, 0.41]
Missing	0.46	[0.39, 0.52]
**Racial and ethnic identity**		
NL White	0.45^c,d,e^	[0.42, 0.47]
NL Native American	0.53 ^f,g^	[0.41, 0.66]
NL Asian/Pacific Islander	0.33^c, f, h, i^	[0.30, 0.36]
NL Black	0.35 ^d^	[0.29, 0.42]
Latina/x/o	0.39^e,g^	[0.38, 0.41]
NL Multiracial	0.42 ^h^	[0.38, 0.46]
Missing	0.47^i^	[0.36, 0.58]
**Sexual Orientation**		
Straight	0.41^j,k^	[0.40, 0.43]
Gay or Lesbian	0.32^j,l^	[0.25, 0.40]
Bisexual	0.42	[0.38, 0.47]
Questioning	0.45	[0.39, 0.51]
Something else	0.46	[0.37, 0.55]
Missing	0.50^k,l^	[0.46, 0.58]
**Gender modality**		
Cisgender	0.42	[0.40, 0.43]
Transgender and gender diverse	0.31	[0.19, 0.43]
Questioning	0.45	[0.35, 0.56]
Missing	0.45	[0.35, 0.56]

Abbreviation: NL = Non‐Latina/x/o.

* Stratified models were run separately for each category. Reported coefficients refer to the main effect of one standard deviation of school climate on sleep duration within each category. Columns with the same letters significantly differ in group comparisons within the same demographic characteristic. NL = Non‐Latina/x/o.

The main effects of all four domains of school climate were positively associated with sleep duration (school safety: *b* = 0.22, *p* < 0.001; caring relationships: *b* = 0.24, *p* < 0.001; meaningful participation: *b* = 0.25, *p* < 0.001; school connectedness: *b* = 0.26, *p* < 0.001). The stratified regression models indicated differences of the effects of these domains on sleep duration across social positions (Table S3, Supporting Materials). While associations between each of the four school climate domains and sleep duration were weaker, on average, for adolescents in 11th and 12th grades compared to adolescents in 9th and 10th grades, differences were more nuanced when considering other social positions. For example, the positive association between caring relationships and sleep duration was attenuated for some racial and ethnic, sexual, and gender minoritized adolescents. In contrast, meaningful participation and school connectedness had smaller benefits on sleep duration particularly among some racial and ethnic minoritized adolescents: sexual and gender minoritized adolescents had benefits on sleep duration that were similar to non‐minoritized adolescents. Notably, the relationships of each of the four school climate domains were smaller for NL Asian and Pacific Islander adolescents compared to NL White adolescents.

**FIGURE 1:**
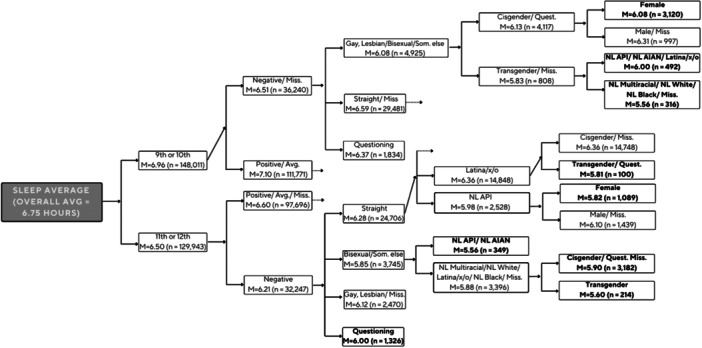
Illustration of a section of the ECHAID tree output. Note: Terminal nodes in bold represent nine of the 10 intersecting groups with the lowest averages of sleep duration. Dotted arrows = Groups continued to split by social positions, but are not presented here due to space limitations. AIAN = American Indian and Alaskan Native, API = Asian and Pacific Islander, Avg = Average, Miss. = Missing, NL = Non‐Hispanic/Latino, Quest. = Questioning gender identity. The full illustration of the output is available upon request.

The ECHAID decision‐tree results include 89 total and 51 terminal nodes. Results for the 10 intersectional groups with the lowest and the highest averages on sleep duration are presented in Tables 3 and 4, respectively (Figure 1 illustrates portions of the decision tree that include nine of the 10 intersectional terminal nodes in Table 3, those with the lowest averages on sleep duration). A pattern emerged in which sexual minoritized adolescents (especially those who were bisexual or something else), NL Asian/Pacific Islanders, those with negative perceptions of school climates, and those in 11th or 12th grades reported the lowest averages of sleep duration. Two intersecting groups reported the lowest average of sleep (sleeping on average 5.56 h per night, or over an hour less than the full sample average): one group was comprised of adolescents in the 9th or 10th grades who perceived their school climate as negative, were gay or lesbian, bisexual, or “something else” for sexual orientation, were transgender or did not report gender modality, and identified as NL White, NL Multiracial, and NL Black. The other group comprised of adolescents in 11th or 12th grades who perceived their school climates as negative, were bisexual or reported “something else” for sexual orientation, and were NL Asian/Pacific Islander, or NL American Indian or Alaska Native. Moreover, transgender adolescents across grades with minoritized sexual orientations and negative perceptions of school climate were represented in three of the shortest sleep groups, averaging about 2 h less than recommended for their age.

**TABLE 3:** Intersectional groups reporting the lowest averages of sleep duration (overall average = 6.75 h).

Mean hours of sleep	Grade	Sex assigned at birth	Racial or ethnic identity	Gender Modality	Sexual orientation	School Climate
5.56 *n* = 316	9th or 10th	All	NL White/NL Multiracial/NL Black	Transgender/Missing	LG/Bisexual/Something else	Negative/Missing
5.56 *n* = 349	11th or 12th	All	NL API/NL AIAN	All	Bisexual/Something else	Negative
5.60 *n* = 214	11th or 12th	All	NL White/NL Black/NL Multiracial/Latina/x/o/Missing	Transgender	Bisexual/Something else	Negative
5.81 *n* = 100	11th or 12th	All	Latina/x/o	Transgender/Questioning	Straight	Negative
5.82 *n* = 1089	11th or 12th	Female	NL API	All	Straight	Negative
5.88 *n* = 666	11th or 12th	Female/Missing	NL API	All	LG/Bisexual	Positive/Average
5.90 *n* = 3182	11th or 12th	All	NL White/NL Black/NL Multiracial/Latina/x/o/Missing	Cisgender/Questioning/Missing	Bisexual/Something else	Negative
6.00 *n* = 1326	11th or 12th	All	All	All	Questioning	Negative
6.00 *n* = 492	9th or 10th	All	NL API/NL AIAN/Latina/x/o	Transgender/Missing	LG/Bisexual/Something else	Negative/Missing
6.08 *n* = 3120	11th or 12th	Female	All	Cisgender/Questioning	LG/Bisexual/Something else	Negative

*Note:* For cells that display “All,” sleep hours averages were not distinguished based on a specific social position (e.g., sex assigned at birth). These groups include participants from all categories within that social position (e.g., yes, no, missing).

Abbreviations: AIAN, American Indian or Alaska Native; API, Asian or Pacific Islander; LG, Lesbian or Gay; NL, Non‐Hispanic/Latina/x/o.

**TABLE 4:** Intersectional groups reporting the highest averages of sleep duration.

Mean hours of sleep	Grade	Sex assigned at birth	Racial or ethnic identity	Gender modality	Sexual orientation	School Climate
7.49 *n* = 18,362	9th or 10th	Male/Missing	All	All	Straight/Missing	Positive
7.38 *n* = 625	9th or 10th	Female	All	All	Missing	Positive
7.24 *n* = 404	9th or 10th	Male/Missing	All	All	Questioning	Positive
7.24 *n* = 15,258	9th or 10th	Female	All	All	Straight/Questioning	Positive
7.14 *n* = 33,392	9th or 10th	Male	All	All	Straight/Missing	Average
7.00 *n* = 931	9th or 10th	Male/Missing	All	All	Questioning	Average
6.96 *n* = 5080	11th or 12th	Male/Missing	NH White/NH AIAN/Missing	All	All	Positive/Missing
6.95 *n* = 31,587	9th or 10th	Female/Missing	All	All	Straight/Missing	Average
6.92 *n* = 826	9th or 10th	Male/Missing	All	All	LG/Bisexual/Something else	Positive
6.88 *n* = 1475	9th or 10th	Female	All	All	LG/Bisexual/Something else	Positive

*Note*: For cells that display “All,” sleep hours averages were not distinguished based on a specific social position (e.g., sex assigned at birth). These groups include participants from all categories within that social position (e.g., yes, no, missing).

Abbreviations: NL, Non‐Hispanic/Latina/x/o; API, Asian or Pacific Islander; AIAN, American Indian or Alaska Native; LG, Lesbian or Gay.

Sleep duration averages were highest especially among adolescents who were in 9th and 10th grades, were straight or questioning their sexual orientation, and perceived their school climates as positive. Notably, even adolescents in the groups with the highest averages for sleep duration were not sleeping the recommended hours of sleep (highest average = 7.49, see Table 4).

### 5.1 Sensitivity Analysis

Given that there is evidence that adolescents with compromised mental health experience not only shorter sleep but also excessive sleep (Liu et al. 2020), and prior studies have shown that negative school climates are associated with compromised mental health (Aldridge and McChesney 2018), it is possible that the association between school climates and sleep duration is not linear. A first set of sensitivity analyses (similar to the linear regression for the main models) was conducted to test whether the association between school climates had the same pattern when excluding participants who reported an average of 10 or more hours of sleep (see Table S4 in Supporting Information for descriptive characteristics of this subsample; see Table S5 for sensitivity analysis). Results indicated no substantial differences in the association between school climate scores and sleep duration between the sensitivity test models (Table S5) and the main models.

In the ECHAID models that excluded participants who slept, on average, 10 or more hours (see Tables S6 and S7 in Supporting Information), when considering the groups that had the highest averages of sleep duration, several intersectional groups in the model were also represented in the main ECHAID model. Results differ somewhat from the main ECHAID model, yet the supplementary model also demonstrates that participants with intersecting minoritized identities (e.g., gender minorities who were also sexual minorities, or racial minorities who were also sexual minorities) were overrepresented among the groups with the lowest averages of sleep duration. Because negative school climates were overrepresented among participants who had the lowest sleep averages, it is possible that the inconsistency between the sensitivity and the main ECHAID models was driven by the exclusion of participants who had 10 or more hours of sleep, suggesting that school climate indeed has a non‐linear association with sleep duration. To test this hypothesis, two logistic regressions were conducted to examine the associations between school climate scores and (1) having insufficient sleep (i.e., sleeping 7 or less hours) and (2) having too much sleep (i.e., sleeping 10 or more hours) controlling for demographic covariates (i.e., grade, sex assigned at birth, race/ethnicity, sexual orientation, gender modality). For these analyses, two dichotomous variables were created representing whether participants had insufficient sleep (i.e., sleeping 7 h or less) or sufficient sleep (i.e., sleeping 8 or 9 h), and whether participants had too much sleep (i.e., sleeping 10 or more hours) or sufficient sleep (i.e., sleeping 8 or 9 h). The first logistic regression examining the associations between school climate scores and insufficient sleep corroborated findings from the linear regression; that is, participants who reported more positive school climates had a lower likelihood of having insufficient sleep (*OR* = 0.60, 99% CI [0.59, 0.61], *p* < 0.001) than having sufficient sleep. The association between school climate scores and having too much sleep revealed a similar pattern found among those who had insufficient sleep: participants who reported more positive school climates were less likely to report sleeping 10 or more hours (*OR* = 0.84, 99% CI [0.79, 0.88], *p* < 0.001) compared to participants who had sufficient sleep (Table S8 in Supporting Information).

## 6 Discussion

Although sleep has a critical role for adolescents' well‐being (Matricciani et al. 2019), research has indicated that there are differences in sleep duration across social positions (Clark et al. 2023; Dai et al. 2020; Levenson et al. 2021; Real et al. 2024; Saelee et al. 2023) and across different contexts (Mayne et al. 2021; Meldrum et al. 2018; Troxel et al. 2017). This study used an intersectionality approach to explore how sleep duration varies for adolescents based on perceptions of school climate and their social positions. Our findings converge with those from Meldrum et al. (2018) indicating consistent associations between school climate and sleep duration: perceiving a negative school climate is associated with shorter sleep among adolescents. Yet, the present study adds to the literature by examining positive aspects of school climate and by showing the extent to which school climate varies based on adolescents' social positions and contributes to differences in sleep duration. More specifically, by utilizing two complementary statistical methods, we demonstrated that positive school climates were protective of sleep duration for some adolescents more than others, and those who perceived their school climates as negative and held multiple intersecting minoritized identities were sleeping much less than others. In addition, our supplementary analyses show that each school climate domain may have unique contributions to sleep duration. Finally, our study reveals that short sleep is widespread among adolescents, such that all groups found in the ECHAID analysis (including those reporting the highest averages of sleep duration) were sleeping less than the recommended 8 h per night on average.

In multiple ways, our results indicate that sleep duration is associated with school climate. First, we found that school climates can be either a risk factor (when perceived as negative) or a protective factor (when perceived as positive) for inadequate sleep duration among adolescents. Specifically, in the ECHAID analysis, a consistent pattern emerged: negative school climates were overrepresented among those who reported the lowest averages of sleep duration, and positive school climates were overrepresented among those who reported the highest averages of sleep duration. Importantly, our sensitivity analysis supported the idea that the relationship between school climate and sleep duration has a U‐shape form, just like the associations between sleep duration and mental health (Liu et al. 2020). In this sense, positive school climate is linked to adequate sleep, and negative school climate is linked to both shorter and too much sleep. These findings underscore that researchers should make distinctions between enough sleep and too much sleep not only when considering sleep duration as a predictor of mental health, but also when considering sleep duration as an outcome.

Second, the linear regressions indicated that one standard deviation increase in the school climate measure was associated with approximately 25 min more sleep per night, on average, among adolescents. While these differences across school climates may seem small, a previous study has shown that sleeping as much as 15 min more is linked to less daytime sleepiness and improved physical health (Stock et al. 2020). This finding brings important considerations for public health: much of the focus on the strategies to improve sleep has considered individual interventions (e.g., sleep hygiene); however, the promotion of better school climate can improve adolescents' sleep, which in turn could boost well‐being (Matricciani et al. 2019) and academic achievement (Hershner 2020) for many adolescents.

Notably, we found that the benefits of positive school climates in sleep duration were not equal among all adolescents: for those who were in higher grades, assigned female at birth, gay or lesbian, transgender, and in some racial and ethnic minoritized groups, the effects of school climate on sleep duration were attenuated. Our supplementary analyses examining the contributions of each school climate domain on sleep duration indicated that racial and ethnic minoritized adolescents, and in particular, NL Asian and Pacific Islander adolescents, experience less benefits of multiple domains of school climate on sleep duration. In addition to racial and ethnic minoritized adolescents, caring relationships at school were not as beneficial for sleep duration for some sexual and gender minoritized adolescents. It is possible that these domains of school climate may not be able to mitigate or protect against the systemic stressors (e.g., racism, cisgenderism, heterosexism) that these adolescents with marginalized identities experience in other contexts. Furthermore, positive school climates may be time‐limited in their protection. A study of racially and ethnically diverse youth found that electronic bullying—which can occur at any moment up until the time of sleep—was associated with shorter sleep duration, while bullying during the school day was not (Sutter et al. 2023), suggesting that non‐school peer interactions may overpower school experiences. Altogether, these findings suggest that these minoritized adolescents face additional challenges that can impact their sleep even in positive school climates.

Methodologically, while the inclusion of multiple interaction terms in linear regressions can be infeasible to analyze and interpret, ECHAID provides readily interpretable results from the interaction of multiple categorical variables. Notably, by utilizing decision‐tree methods (i.e., ECHAID), which have been recommended for research using an intersectionality approach (Bauer et al. 2021), we were able to extend findings from the linear regressions. For example, although the linear regressions indicated that school climate was linked to smaller differences in sleep duration, results from the ECHAID analysis showed that these differences were much larger among multiply minoritized adolescents. There was a difference of approximately two fewer hours per night among multiply minoritized adolescents who had negative school climates compared to the group who reported the highest average of sleep duration (and more than 1 h than the overall sample average), suggesting that adolescents at the intersection of multiple minoritized social positions may be burdened with unique stressors that are beyond school climate. Prior studies have reported that multiply minoritized adolescents are particularly vulnerable to multiple forms of bias‐based bullying, which can also occur outside school environments (Gower et al. 2023; Real et al. 2024). These adverse experiences may activate stress responses that can not only biologically and psychologically affect sleep (Gibbs and Fusco 2023; Schacter 2021) but also lead to psychological disorders (Eisenberg et al. 2024; Kreski and Keyes 2022), which in turn can lead to sleep disturbances. Alternatively, because these adolescents experience negative climates in their school (which may include less caring relationships), they may seek to distract themselves from the negative experiences at school or even seek more connections and support in time outside school, and that could affect their sleep. For example, minoritized adolescents often use social media as a way to find emotional support and validation for their experiences (Selkie et al. 2020). Adolescents also tend to use social media as a means to distract themselves (Siebers et al. 2022). In turn, the excessive use of social media (and electronic devices, in general) affects adolescents' sleep (Ricketts et al. 2022; Scott and Woods 2018).

Among adolescents who perceived their school climate as negative, those who were transgender and had a sexual minoritized identity, and those who were NL Asian/Pacific Islander slept disproportionately less. These findings align with other studies showing that these groups are at risk for sleep deprivation (Dai et al. 2020; Galland et al. 2020; Levenson et al. 2021). Shorter sleep among these groups has been linked to experiences of discrimination, such as bullying experiences based on race, gender, and/or sexual orientation (Real et al. 2024; Xie et al. 2021; Yip et al. 2024). Yet, it was noticeable that although NL Asian and Pacific Islander adolescents reported positive climates in somewhat similar proportions compared to NL White adolescents (Table S1), the contributions of positive school climates (and each school climate domain) to sleep duration was substantially smaller for NL Asian and Pacific Islanders compared to NL White adolescents. Additional studies are needed to understand possible reasons for such smaller associations despite perceiving positive climates.

### 6.1 Strengths and Limitations

The strengths of this study include the examination of a large population‐based sample that included participants with a variety of sexual orientations, racial and ethnic identities, and gender identities, conferring enough power to study sleep duration for adolescents at the intersection of minoritized social positions. In this regard, while using a robust model specification (e.g., a minimum terminal node size of 100), we were able to identify groups who experience short sleep even when they were present in relatively small numbers in the sample; they are often not included or are neglected in other studies. Unlike other studies, we use numerous items measuring different dimensions of school climate providing a more global assessment of this construct. Also, while presenting similar findings, the application of two statistical methods allowed us to make further interpretations of our findings.

Nevertheless, this study is not without limitations. First, the CHKS is a school‐based sample and does not include adolescents who did not attend school when data collection occurred. Given that adolescents with poor sleeping patterns or with more negative school climates are more likely to be absent from school (Hysing et al. 2015; Russell et al. 2014), our results may underestimate the link between school climate and sleep for adolescents. Second, we are unable to determine causal associations because the data are cross‐sectional—that is, adolescents who routinely get less sleep (for reasons unrelated to school) may connect and participate less due to tiredness, rather than—or in addition to—poor school climate leading to poorer sleep. Third, self‐reported data may be subject to bias (e.g., social desirability, recall bias). However, self‐reports of sleep patterns are viable for samples of this size, and self‐reported sleep tends to be highly correlated with objective measures (e.g., actigraphy) (Nascimento‐Ferreira et al. 2016). Fourth, it is not possible to determine whether some adolescents in this sample could be naturally short sleepers (Curtis et al. 2018); naturally short sleepers may not experience negative implications due to continuous short sleep. Lastly, the ways social positions are measured in the CHKS have notable limitations. For example, participants' response options for gender modality did not include nonbinary or additional relevant terms, and the measure of sexual orientation does not include a wide range of labels adolescents may use, such as pansexual or queer. Similarly, by asking “What is your sex?” to assess participants' sex assigned at birth, it is possible that some adolescents based their responses on their gender identity.

While the inclusion of data with missing responses in our analyses was intentional and exploratory, the interpretation of intersecting groups that include participants who had missing data is challenging. Rates of missingness were relatively low across the items assessing sex assigned at birth (2.5%), racial and ethnic identity (0.9%), sexual orientation (4.0%), and gender modality (3.6%). Notably, the majority of the missingness (75%–82%) present in the sexual orientation and gender modality measures is from participants who selected the *Decline to respond* option. In terms of gender modality, for example, it is possible that these groups include adolescents who are cisgender, and adolescents whose identities were not an option in the CHKS survey (e.g., nonbinary). In other words, these adolescents who had missing data are not a monolithic group and the reasons for the presence of participants who had missing data among those with the highest and lowest averages of sleep duration are likely not the same. More studies are needed to explain these associations.

### 6.2 Implications

Our study documents that school climate and each of its four domains (i.e., school safety, caring relationships, school connectedness, and meaningful participation) are associated with longer sleep duration for adolescents. Given the importance of sleep for adolescents' well‐being and academic performance, our study points to a critical need for school staff and policymakers to develop strategies to promote better school climates for all students. For example, schools may improve school safety with the inclusion of enumerated anti‐bullying policies and processes that make school districts accountable for fostering safe schools (e.g., regular evaluations of school climates) (Hatzenbuehler et al. 2015; Lacoe 2020; Real et al. 2024). While these implementations may be beneficial for all students, they may be particularly beneficial for adolescents at the intersection of minoritized social positions given that these adolescents are more likely to experience bullying and perceive school climates as negative (Gower et al. 2023; Ioverno and Russell 2022). Furthermore, our findings demonstrate that transgender and sexual minoritized adolescents are particularly vulnerable to shorter sleep durations; the presence of gender‐sexuality alliances at school may provide a network of support and caring relationships especially for these adolescents. Prior studies have shown that gender‐sexuality alliances are associated with better school climates (Ioverno et al. 2016) and greater well‐being among adolescents (Krantz et al. 2024). Schools can also improve students' experiences by promoting a sense of connection with the school. Students feel more connected with their schools when these schools have inclusive curriculum, staff with a diversity of backgrounds, and enumerated anti‐bullying policies (Kosciw et al. 2022). Lastly, schools can offer a variety of positive youth development opportunities to encourage students to engage with their schools. Students who participate in meaningful ways in their school have higher academic attainment (Thapa et al. 2013). Activities that promote meaningful participation at school may include community service, involvement in social and government processes, and opportunities to provide feedback on the learning environment.

## 7 Conclusions

Adolescence is a period of risk for insufficient sleep. Such higher risk may be due to the increasing demands from school (e.g., homework, extracurricular activities), excessive use of electronic devices, and early school start times that conflict with a delayed circadian rhythm at this age (Gariepy et al. 2020; Ricketts et al. 2022). Findings from this study show that, in addition to these factors, school climate may play a role in sleep duration among adolescents. Thus, strategies that promote better school climates should be an avenue for future interventions and can impact a large number of students (Singla et al. 2021). Our study finds evidence that adolescents holding multiple minoritized social positions experience additional challenges that can affect sleep. Future research should continue to examine how the intersection of social positions is associated with adolescents' well‐being and the underlying mechanisms that are possibly involved.

## Author Contributions

A.G.R. conceived the study, participated in its design, conducted statistical analyses, participated in the interpretation of the findings, and drafted the manuscript. S.T.R. and M.E.E. conceived the study, participated in its design and interpretation of findings, and helped draft the manuscript. B.T.G., G.N.R., B.P., and S.E.L. helped draft the manuscript. All authors read and approved the final manuscript.

## Ethics Statement

The University of Minnesota's Institutional Review Board determined that this study was exempt from review, due to the use of existing, anonymous data.

## Conflicts of Interest

The authors declare no conflicts of interest.

## Supporting information

Supporting Materials 1020.
