# Supplementary material for: School Climate and Sleep Duration Among Adolescents at the Intersection of Multiple Social Positions
Source: J Adolesc. 2026 Jan 15;98(3):929–41. doi: 10.1002/jad.70105 (PMC13044870; doi:10.1002/jad.70105)
Supplement: Supplementary file 1 — Supporting Materials 1020. [file JAD-98-929-s001.docx]

**Supplementary Materials**

| **Table S1.** Nonstandardized averages for each school climate domain across participants. (N=277,954) | | | | | | |  |  |  |  |
| --- | --- | --- | --- | --- | --- | --- | --- | --- | --- | --- |
|  |  | Average of school safety (SD) | *F* | Average of school caring relationships (SD) | *F* | Average of school connectedness (SD) | *F* | Average of school meaningful participation (SD) | *F* |  |
| **Range** |  | 0-4 |  | 0-3 |  | 0-4 |  | 0-4 |  |  |
| **Grade** |  |  |  |  |  |  |  |  |  |  |
| 9th or 10th |  | 2.60 (0.86) | 1.8 | 1.81 (0.78)^a^ | 409.9*** | 2.52 (0.85)^a^ | 721.9*** | 1.45 (0.77)^a^ | 19.8*** |  |
| 11th or 12th |  | 2.61 (0.86) |  | 1.87 (0.80)^a^ |  | 2.43 (0.87)^a^ |  | 1.46 (0.79)^a^ |  |  |
| **Sex assigned at birth** |  |  |  |  |  |  |  |  |  |  |
| Male |  | 2.64 (0.89)^a,b^ |  | 1.84 (0.79)^b^ |  | 2.53 (0.87)^b,c^ |  | 1.42 (0.79)^b,c^ |  |  |
| Female |  | 2.58 (0.83)^a,c^ | 235.2*** | 1.84 (0.79)^c^ | 22.6*** | 2.44 (0.84)^b,d^ | 440.0*** | 1.49 (0.76)^b,d^ | 272.7*** |  |
| Missing |  | 2.53 (0.91)^b,c^ |  | 1.77 (0.83)^b,c^ |  | 2.39 (0.92)^c,d^ |  | 1.45 (0.81)^c,d^ |  |  |
| **Racial and ethnic identity** |  |  |  |  |  |  |  |  |  |  |
| NL Native American |  | 2.59 (0.93)^d,e,f^ |  | 1.79 (0.84)^d,e,f^ |  | 2.42 (0.97)^e,f,g^ |  | 1.48 (0.83)^e^ |  |  |
| NL Asian/Pacific Islander |  | 2.69 (0.81)^d,g,h,i,j,k^ |  | 1.85 (0.75)^d,g,h,i^ |  | 2.55 (0.81)^e,h,i,j,k,l^ |  | 1.53 (0.78)^f,g,h,i,j^ |  |  |
| NL Black |  | 2.47 (0.92)^e,g,l,m,n,o^ | 487.3*** | 1.88 (0.82)^e,j,k,l,m^ | 481.5*** | 2.27 (0.94)^f,h,m,n,o,p^ | 508.2*** | 1.49 (0.80)^f,k,l^ | 100.8*** |  |
| Latina/x/o |  | 2.54 (0.86)^h,m,p,q,r^ |  | 1.77 (0.80)^g,j,n,o^ |  | 2.42 (0.85)^i,m,q,r^ |  | 1.43 (0.76)^g,l,m,n^ |  |  |
| NL White |  | 2.75 (0.86)^f,i,n,p,s,t^ |  | 1.98 (0.78)^f,h,k,n,p,q^ |  | 2.62 (0.87)^g,j,n,q,s,t^ |  | 1.48 (0.81)^h,m,o^ |  |  |
| NL Multiracial |  | 2.57 (0.88)^j,n,q,s^ |  | 1.84 (0.79)^l,o,p,r^ |  | 2.46 (0.88)^k,o,r,s^ |  | 1.41 (0.78)^e,i,k,n,o^ |  |  |
| Missing |  | 2.60 (0.89)^k,o,r,t^ |  | 1.77 (0.82)^i,m,q,r^ |  | 2.42 (0.91)^l,p,t^ |  | 1.45 (0.82)^j^ |  |  |
| **Sexual orientation** |  |  |  |  |  |  |  |  |  |  |
| Straight |  | 2.65 (0.85)^u,v,w,x,y^ |  | 1.85 (0.78)^s,t,u,v,w^ |  | 2.52 (0.85)^u,v,w,x,y^ |  | 1.47 (0.77)^p,q,r,s^ |  |  |
| Gay or Lesbian |  | 2.38 (0.94)^u,z,aa,ab^ |  | 1.79 (0.86)^s,x,y,z^ |  | 2.30 (0.93)^u,z,aa^ |  | 1.36 (0.81)^p,t,u,v^ |  |  |
| Bisexual |  | 2.40 (0.88)^v,ac,ad,ae^ | 603.4*** | 1.75 (0.83)^t,x,aa,ab^ | 192.6*** | 2.28 (0.86)^v,ab,ac^ | 526.9*** | 1.31 (0.77)^q,t,x,y^ | 237.1*** |  |
| Questioning |  | 2.51 (0.86)^w,z,ac,af,ag^ |  | 1.76 (0.80)^u,ac,ad^ |  | 2.37 (0.86)^w,z,ab,ad,ae^ |  | 1.37 (0.76)^r,x,w,z^ |  |  |
| Something else |  | 2.30 (0.97)^x,aa,ad,af,ah^ |  | 1.68 (0.85)^v,y,aa,ac^ |  | 2.24 (0.93)^x,aa,ad,af^ |  | 1.30 (0.81)^s,u,w,aa^ |  |  |
| Missing |  | 2.45 (0.92)^y,ab,ae,af,ah^ |  | 1.74 (0.84)^w,z,ab,ad^ |  | 2.32 (0.90)^y,ac,ae,af^ |  | 1.46 (0.79)^v,y,z,aa^ |  |  |
| **Gender modality** |  |  |  |  |  |  |  |  |  |  |
| Cisgender |  | 2.62 (0.85)^ai,aj,ak^ |  | 1.85 (0.79)^ae,af,ag^ |  | 2.50 (0.85)^ag,ah,ai^ |  | 1.46 (0.77)^ab,ac,ad^ |  |  |
| Transgender |  | 2.12 (1.05)^ai,al,am^ | 732.8*** | 1.54 (0.90)^ae,ah,ai^ | 431.1*** | 2.02 (1.02)^ag,aj,ak^ | 651.7*** | 1.23 (0.84)^ab,ae,af^ | 130.6*** |  |
| Questioning |  | 2.27 (0.98)^aj,al,an^ |  | 1.60 (0.84)^af,ah,aj^ |  | 2.19 (0.95)^af,aj,al^ |  | 1.29 (0.79)^ac,ae,ag^ |  |  |
| Missing |  | 2.38 (0.97)^ak,am,an^ |  | 1.65 (0.83)^ag,ai,aj^ |  | 2.26 (0.95)^ai,ak,al^ |  | 1.49 (0.82)^ad,af,ag^ |  |  |
| Note: Percent of missing calculated based on full N. All valid response options are shown as the valid percent (excludes missing values). NL = Non-Latina/x/o. Columns with the same letters significantly differ between group comparisons. *p<.05, **p<.01, ***p<.001 | | | | | | | | | |  |
|  |  |  |  |  |  |  |  |  |  |  |

| **Table S2.** Distribution of participants across school climate quartiles. | | | | |
| --- | --- | --- | --- | --- |
|  |  |  |  |  |
| **Characteristic** |  | Negative - N (%) | On average - N (%) | Positive - N (%) |
| **Grade** |  |  |  |  |
| 9th or 10th |  | 36,203 (24.5) | 74,821 (50.6) | 36,950 (25.0) |
| 11th or 12th |  | 32,247 (24.8) | 64,886 (50.0) | 32,768 (25.2) |
| **Sex assigned at birth** |  |  |  |  |
| Male |  | 32,432 (24.1) | 67,205 (49.9) | 35,160 (26.1) |
| Female |  | 34,048 (25.0) | 69,118 (50.8) | 32,929 (24.2) |
| Missing |  | 1,970 (28.2) | 3,384 (48.5) | 1,629 (23.3) |
| **Racial and ethnic identity** |  |  |  |  |
| NL Native American |  | 559 (27.6) | 936 (46.3) | 529 (26.1) |
| NL Asian/Pacific Islander |  | 6,814 (20.3) | 17,668 (52.7) | 9,053 (27.0) |
| NL Black |  | 2,414 (27.8) | 4,249 (48.9) | 2,028 (23.3) |
| Latina/x/o |  | 40,393 (27.1) | 75,709 (50.9) | 32,704 (22.0) |
| NL White |  | 11,725 (19.7) | 28,626 (48.0) | 19,240 (32.3) |
| NL Multiracial |  | 5,835 (25.8) | 11,259 (49.8) | 5,519 (24.4) |
| Missing |  | 710 (27.2) | 1,260 (48.2) | 645 (24.7) |
| **Sexual orientation** |  |  |  |  |
| Straight |  | 52,241 (22.9) | 115,512 (50.7) | 60,042 (26.4) |
| Gay or Lesbian |  | 1,726 (32.2) | 2,545 (47.5) | 1,089 (20.3) |
| Bisexual |  | 6,214 (33.6) | 9,035 (48.9) | 3,245 (17.6) |
| Questioning |  | 3,158 (29.5) | 5,337 (49.8) | 2,230 (20.8) |
| Something else |  | 1,580 (36.8) | 1,953 (45.5) | 764 (17.8) |
| Missing |  | 3,531 (31.5) | 5,325 (47.5) | 2,348 (21.0) |
| **Gender modality** |  |  |  |  |
| Cisgender |  | 62,391 (23.9) | 132,441 (50.6) | 66,783 (25.5) |
| Transgender |  | 1,238 (47.1) | 1,017 (38.7) | 371 (14.1) |
| Questioning |  | 1,403 (39.4) | 1,616 (45.4) | 543 (15.2) |
| Missing |  | 3,418 (33.9) | 4,633 (46.0) | 2,021 (20.1) |
| Note: NL = Non-Latina/x/o. | | | | |

**Sensitivity analysis**

| **Table S3.** Multiple linear regressions examining the main effects of each of the four school climate domains on sleep duration stratified by social positions. | | | | | | | | | | | | | |
| --- | --- | --- | --- | --- | --- | --- | --- | --- | --- | --- | --- | --- | --- |
|  |  | **School safety** | |  | **Caring relationships** | |  | **School connectedness** | |  | **School meaningful participation** | |  |
|  |  | ***b*** | ***99% CI*** |  | ***b*** | ***99% CI*** |  | ***b*** | ***99% CI*** |  | ***b*** | ***99% CI*** |  |
| Grade |  |  |  |  |  |  |  |  |  |  |  |  |  |
| 9th or 10th Grade |  | 0.24^a^ | [0.24, 0.26] |  | 0.29^a^ | [0.28, 0.30] |  | 0.30^a^ | [0.29, 0.32] |  | 0.31^a^ | [0.30, 0.32] |  |
| 11th or 12th Grade |  | 0.19^a^ | [0.18, 0.20] |  | 0.18^a^ | [0.17, 0.19] |  | 0.22^a^ | [0.21, 0.23] |  | 0.18^a^ | [0.17, 0.20] |  |
| Sex Assigned at Birth |  |  |  |  |  |  |  |  |  |  |  |  |  |
| Male |  | 0.22 | [0.21, 0.23] |  | 0.26^b^ | [0.25, 0.27] |  | 0.27 | [0.26, 0.28] |  | 0.29^b^ | [0.27, 0.30] |  |
| Female |  | 0.22 | [0.21, 0.23] |  | 0.22^b^ | [0.21, 0.23] |  | 0.26 | [0.25, 0.27] |  | 0.21^b^ | [0.20, 0.23] |  |
| Missing |  | 0.24 | [0.19, 0.29] |  | 0.26 | [0.21, 0.32] |  | 0.30 | [0.25, 0.36] |  | 0.27 | [0.21, 0.33] |  |
| Racial and ethnic identity |  |  |  |  |  |  |  |  |  |  |  |  |  |
| NL White |  | 0.24^b,c^ | [0.22, 0.26] |  | 0.28^c,d,e^ | [0.27, 0.30] |  | 0.29^c,d^ | [0.27, 0.31] |  | 0.28^c^ | [0.26, 0.30] |  |
| NL Native American |  | 0.31^d,e^ | [0.22, 0.41] |  | 0.33^f^ | [0.23, 0.43] |  | 0.36 | [0.26, 0.45] |  | 0.34^d^ | [0.23, 0.45] |  |
| NL Asian/Pacific Islander |  | 0.20^b,d^ | [0.18, 0.22] |  | 0.19^c,f,g^ | [0.17, 0.22] |  | 0.24^c^ | [0.21, 0.26] |  | 0.18^c, d, e, f^ | [0.16, 0.21] |  |
| NL Black |  | 0.20^c,e^ | [0.12, 0.21] |  | 0.20^d^ | [0.15, 0.25] |  | 0.22^d^ | [0.17, 0.26] |  | 0.27 | [0.22, 0.33] |  |
| Latina/x/o |  | 0.22 | [0.21, 0.23] |  | 0.23^e^ | [0.22, 0.24] |  | 0.26 | [0.25, 0.27] |  | 0.25^e^ | [0.23, 0.26] |  |
| NL Multiracial |  | 0.23 | [0.20, 0.25] |  | 0.25 | [0.22, 0.28] |  | 0.28 | [0.24, 0.31] |  | 0.27^f^ | [0.23, 0.30] |  |
| Missing |  | 0.27 | [0.19, 0.35] |  | 0.32^g^ | [0.23, 0.40] |  | 0.30 | [0.21, 0.39] |  | 0.30 | [0.20, 0.39] |  |
| Sexual Orientation |  |  |  |  |  |  |  |  |  |  |  |  |  |
| Straight |  | 0.21^f,g^ | [0.21, 0.22] |  | 0.24^h,i^ | [0.23, 0.25] |  | 0.26 | [0.25, 0.27] |  | 0.25 | [0.24, 0.26] |  |
| Gay or Lesbian |  | 0.21 | [0.15, 0.26] |  | 0.15^h,j^ | [0.09, 0.21] |  | 0.20 | [0.13, 0.26] |  | 0.20 | [0.13, 0.26] |  |
| Bisexual |  | 0.22 | [0.19, 0.25] |  | 0.22^k^ | [0.19, 0.25] |  | 0.28 | [0.24, 0.31] |  | 0.22 | [0.19, 0.26] |  |
| Questioning |  | 0.25 | [0.21, 0.29] |  | 0.25 | [0.21, 0.29] |  | 0.29 | [0.24, 0.33] |  | 0.25 | [0.20, 0.30] |  |
| Something else |  | 0.29^f^ | [0.23, 0.36] |  | 0.22 | [0.15, 0.30] |  | 0.31 | [0.23, 0.38] |  | 0.27 | [0.19, 0.35] |  |
| Missing |  | 0.29^g^ | [0.25, 0.33] |  | 0.31^i,j,k^ | [0.27, 0.36] |  | 0.30 | [0.26, 0.35] |  | 0.33 | [0.28, 0.38] |  |
| Gender modality |  |  |  |  |  |  |  |  |  |  |  |  |  |
| Cisgender |  | 0.22^h^ | [0.21, 0.23] |  | 0.24^l,m^ | [0.23, 0.24] |  | 0.26 | [0.26, 0.27] |  | 0.25^g^ | [0.24, 0.26] |  |
| Transgender |  | 0.22 | [0.14, 0.30] |  | 0.12^l,n,o^ | [0.03, 0.21] |  | 0.21 | [0.12, 0.30] |  | 0.17 | [0.06, 0.27] |  |
| Questioning Gender identity |  | 0.23 | [0.16, 0.31] |  | 0.32^n^ | [0.24, 0.40] |  | 0.25 | [0.17, 0.33] |  | 0.24 | [0.15, 0.33] |  |
| Missing |  | 0.30^h^ | [0.25, 0.34] |  | 0.34^m,o^ | [0.29, 0.38] |  | 0.30 | [0.25, 0.35] |  | 0.33^g^ | [0.27, 0.38] |  |
| Note: *Stratified models were run separately for each category. Reported coefficients refer to the main effects of each of the four school climate domains on sleep duration by each category of social position. Columns with the same letters significantly differ in group comparisons within the same category. | | | | | | | | | | | | | |

|  |
| --- |

| **Table S4.** Sample sociodemographic characteristics among participants who had 10 or more hours of sleep (n = 6,953) | | |
| --- | --- | --- |
|  |  |  |
| **Characteristic** |  | N (%) |
| **Grade** |  |  |
| 9th or 10th |  | 4,694 (67.5) |
| 11th or 12th |  | 2,259 (32.5) |
| **Sex assigned at birth** |  |  |
| Male |  | 3,930 (58.3) |
| Female |  | 2,811 (41.7) |
| Missing |  | 212 (3.1) |
| **Racial and ethnic identity** |  |  |
| NL Native American |  | 82 (1.2) |
| NL Asian/Pacific Islander |  | 503 (7.4) |
| NL Black |  | 330 (4.8) |
| Latina/x/o |  | 4,036 (59.0) |
| NL White |  | 1,283 (18.8) |
| NL Multiracial |  | 605 (8.9) |
| Missing |  | 114 (1.6) |
| **Sexual orientation** |  |  |
| Straight |  | 5,370 (84.5) |
| Gay or Lesbian |  | 146 (2.3) |
| Bisexual |  | 368 (5.8) |
| Questioning |  | 304 (4.8) |
| Something else |  | 169 (2.7) |
| Missing |  | 596 (8.6) |
| **Gender modality** |  |  |
| Cisgender |  | 6,020 (95.0) |
| Transgender |  | 132 (2.1) |
| Questioning |  | 182 (2.9) |
| Missing |  | 619 (8.9) |
| Note: NL = Non-Latina/x/o. Percent of missing calculated based on full N. All valid response options are shown as the valid percent (excludes missing values). | | |

| **Table S5.** Multiple linear regressions examining the effects of social positions and school climate perceptions on sleep duration excluding participants who reported 10 hours or more. | | | | | | |
| --- | --- | --- | --- | --- | --- | --- |
|  | **Main Effects** | | |  | **Main effects for school climate in models stratified by social positions*** | |
|  | ***b*** | ***99% CI*** | ***p*** |  | ***b*** | ***99% CI*** |
|  |  |  |  |  |  |  |
| School Climate (Mean of standardized items) | **0.42** | [0.41, 0.43] | **<.001** |  |  |  |
| Grade |  |  |  |  |  |  |
| 9th or 10th Grade | (ref) |  |  |  | 0.49^a^ | [0.48, 0.51] |
| 11th or 12th Grade | **-0.42** | [-0.43, -0.41] | **<.001** |  | 0.34^a^ | [0.33, 0.35] |
| Sex Assigned at Birth |  |  |  |  |  |  |
| Male | (ref) |  |  |  | 0.45^b^ | [0.43, 0.46] |
| Female | **-0.16** | [-0.17, -0.15] | **<.001** |  | 0.39^b^ | [0.38, 0.41] |
| Missing | **-0.11** | [-0.15, -0.07] | **<.001** |  | 0.47 | [0.40, 0.53] |
| Racial and ethnic identity |  |  |  |  |  |  |
| NL White | (ref) |  |  |  | 0.47^c,d,e^ | [0.45, 0.49] |
| NL Native American | -0.21 | [-0.28, -0.13] | **<.001** |  | 0.51^f^ | [0.39, 0.63] |
| NL Asian/Pacific Islander | **-0.10** | [-0.18, -0.02] | **.002** |  | 0.35^c,f,g,h,i^ | [0.32, 0.38] |
| NL Black | **0.06** | [-0.02, 0.13] | **.052** |  | 0.38^d^ | [0.32, 0.44] |
| Latina/x/o | 0.05 | [-0.02, 0.13] | .073 |  | 0.4^e,g^ | [0.40, 0.43] |
| NL Multiracial | **-0.07** | [-0.15, -0.01] | **.017** |  | 0.46^h^ | [0.42, 0.49] |
| Missing | 0.06 | [-0.04, 0.16] | .112 |  | 0.49^i^ | [0.39, 0.60] |
| Sexual Orientation |  |  |  |  |  |  |
| Straight | (ref) |  |  |  | 0.41^j^ | [0.40, 0.43] |
| Gay or Lesbian | **-0.33** | [-0.38, -0.28] | **<.001** |  | 0.36^k^ | [0.29, 0.44] |
| Bisexual | **-0.37** | [-0.40, -0.35] | **<.001** |  | 0.43 | [0.39, 0.47] |
| Questioning | **-0.18** | [-0.21, -0.14] | **<.001** |  | 0.45 | [0.40, 0.51] |
| Something else | **-0.40** | [-0.46, -0.35] | **<.001** |  | 0.49 | [0.40, 0.58] |
| Missing | **-0.04** | [-0.08, -0.01] | **.002** |  | 0.52^j,k^ | [0.47, 0.57] |
| Gender modality |  |  |  |  |  |  |
| Cisgender | (ref) |  |  |  | 0.42^l^ | [0.40, 0.43] |
| Transgender and gender diverse | **-0.38** | [-0.45, -0.31] | **<.001** |  | 0.39 | [0.28, 0.50] |
| Questioning | **-0.14** | [-0.20, -0.08] | **<.001** |  | 0.47 | [0.38, 0.57] |
| Missing | **-0.09** | [-0.13, -0.05] | **<.001** |  | 0.53^l^ | [0.47, 0.58] |
| Note: *Stratified models were run separately for each category. Reported coefficients refer to the main effects of school climate on sleep duration by each category. Columns with the same letters significantly differ in group comparisons within the same category. | | | | | | |
|  |  |  |  |  |  |  |

Table S6. Intersectional groups reporting the lowest averages of sleep duration excluding participants who reported 10 hours or more.

| Mean hours of sleep | Grade | Sex assigned at birth | Racial and ethnic identity | Gender Modality | Sexual orientation | School Climate |
| --- | --- | --- | --- | --- | --- | --- |
| 5.52 (Node: 91)  *n* = 307 | 11^th^ or 12^th^ | All | NL API/ NL AIAN | Cisgender/ Questioning | LG/ Bisexual | Negative |
| 5.53 (Node: 22)  *n* = 646 | All | All | All | Transgender/ Questioning/ Missing | Something else | Negative |
| 5.67 (Node: 18)  *n* = 903 | All | All | All | Transgender/ Missing | LG/ Bisexual | Negative |
| 5.73 (Node: 47)  *n* = 623 | 11^th^ or 12^th^ | All | NL API/ NL AIAN | All | Questioning/ Missing | Negative |
| 5.76 (Node: 49)  *n* = 598 | All | Female | All | Cisgender | Something else | Negative |
| 5.79 (Node: 85)  *n* = 1,079 | 11^th^ or 12^th^ | Female | NL API | All | Straight | Negative |
| 5.86 (Node: 62)  *n* = 663 | 11^th^ or 12^th^ | Female/ Missing | NL API | All | LG/ Bisexual | Positive/ Average |
| 5.87 (Node: 96)  *n* = 133 | 11^th^ or 12^th^ | Male/ Missing | All | Cisgender | Something else | Negative |
| 5.87 (Node: 92)  *n* = 3,121 | 11^th^ or 12^th^ | All | NL White/ NL Black/ NL Multiracial/ Latina/x/o/ Missing | Cisgender/ Questioning | LG/ Bisexual | Negative |
| 5.92 (Node: 90)  *n* = 1,122 | 9^th^ or 10^th^ | All | NL White/ NL AIAN/ NL Multiracial/ Missing | Cisgender/ Questioning | LG/ Bisexual | Negative |

Table S7. Intersectional groups reporting the highest averages of sleep duration excluding participants who reported 10 hours or more.

| Mean hours of sleep | Grade | Sex assigned at birth | Racial and ethnic identity | Transgender identity | Sexual orientation | School Climate |
| --- | --- | --- | --- | --- | --- | --- |
| 7.37 (Node: 58)  *n* = 16,911 | 9^th^ or 10^th^ | Male/ Missing | All | All | Straight | Positive |
| 7.16 (Node: 57)  *n* = 14,007 | 9^th^ or 10^th^ | Female | All | All | Straight | Positive |
| 7.13 (Node: 29)  *n* = 2,307 | 9^th^ or 10^th^ | All | All | All | Questioning/ Missing | Positive |
| 7.05 (Node: 52)  *n* = 31,176 | 9^th^ or 10^th^ | Male | All | All | Straight | Average |
| 6.91 (Node: 74)  *n* = 4,694 | 11^th^ or 12^th^ | Male/ Missing | NL White | All | All | Positive |
| 6.88 (Node: 51)  *n* = 29,157 | 9^th^ or 10^th^ | Female/ Missing | All | All | Straight | Average |
| 6.86 (Node: 56)  *n* = 845 | 9^th^ or 10^th^ | Male | All | All | Questioning | Average |
| 6.86 (Node: 25)  *n* = 2,793 | 9^th^ or 10^th^ | All | All | All | Missing | Average |
| 6.84 (Node: 59)  *n* = 1,893 | 9^th^ or 10^th^ | All | All | Cisgender | LG/ Bisexual/ Something else | Positive |
| 6.78 (Node: 76)  *n* = 14,077 | 11^th^ or 12^th^ | All | NL AIAN/ Latina/x/o/ Missing | All | Straight/ Missing | Positive |

| **Table S8.** Logistic regressions examining the associations between school climate perceptions and insufficient sleep and sleeping too much. | | | | | | | | |
| --- | --- | --- | --- | --- | --- | --- | --- | --- |
|  | **Insufficient sleep** | | |  | **Too much sleep** | | |  |
|  | ***OR*** | ***99% CI*** | ***p*** |  | ***OR*** | ***99% CI*** | ***p*** |  |
|  |  |  |  |  |  |  |  |  |
| School Climate (Mean of standardized items) | **0.60** | [0.59, 0.61] | **<.001** |  | **0.84** | **[0.79, 0.88]** | **<.001** |  |
| Grade |  |  |  |  |  |  |  |  |
| 9th or 10th Grade | (ref) |  |  |  | (ref) |  |  |  |
| 11th or 12th Grade | **1.99** | [1.94, 2.03] | **<.001** |  | **0.84** | [0.79, 0.90] | **<.001** |  |
| Sex Assigned at Birth |  |  |  |  |  |  |  |  |
| Male | (ref) |  |  |  | (ref) |  |  |  |
| Female | **1.32** | [1.29, 1.35] | **<.001** |  | **0.84** | [0.78, 0.90] | **<.001** |  |
| Missing | **1.14** | [1.06, 1.23] | **<.001** |  | 1.07 | [0.88, 1.29] | .404 |  |
| Racial and ethnic identity |  |  |  |  |  |  |  |  |
| NL White | (ref) |  |  |  | (ref) |  |  |  |
| NL Native American | 1.62 | [1.42, 1.84] | **<.001** |  | **0.51** | [0.37, 0.71] | **<.001** |  |
| NL Asian/Pacific Islander | **1.21** | [1.05, 1.39] | **.001** |  | 1.17 | [0.83, 1.65] | .226 |  |
| NL Black | **1.05** | [0.92, 1.19] | .354 |  | 0.74 | [0.55, 1.01] | .012 |  |
| Latina/x/o | 1.11 | [0.97, 1.26] | .040 |  | **0.60** | [0.44, 0.82] | **<.001** |  |
| NL Multiracial | **1.25** | [1.10, 1.43] | **<.001** |  | 0.77 | [0.56, 1.06] | .038 |  |
| Missing | 0.97 | [0.82, 1.15] | .633 |  | 0.89 | [0.60, 1.33] | .455 |  |
| Sexual Orientation |  |  |  |  |  |  |  |  |
| Straight | (ref) |  |  |  | (ref) |  |  |  |
| Gay or Lesbian | **1.46** | [1.33, 1.60] | **<.001** |  | **1.30** | [1.02, 1.66] | **.005** |  |
| Bisexual | **1.56** | [1.48, 1.65] | **<.001** |  | **1.26** | [1.08, 1.47] | **<.001** |  |
| Questioning | **1.25** | [1.18, 1.33] | **<.001** |  | **1.34** | [1.14, 1.59] | **<.001** |  |
| Something else | **1.45** | [1.31, 1.61] | **<.001** |  | **1.93** | [1.53, 2.43] | **<.001** |  |
| Missing | 0.94 | [0.88, 1.00] | .012 |  | **1.59** | [1.37, 1.84] | **<.001** |  |
| Gender modality |  |  |  |  |  |  |  |  |
| Cisgender | (ref) |  |  |  | (ref) |  |  |  |
| Transgender and gender diverse | **1.40** | [1.22, 1.62] | **<.001** |  | **2.72** | [2.07, 3.58] | **<.001** |  |
| Questioning | 1.02 | [0.91, 1.14] | .615 |  | **2.27** | [1.80, 2.85] | **<.001** |  |
| Missing | **0.92** | [0.86, 0.99] | **.003** |  | **1.99** | [1.72, 2.30] | **<.001** |  |

Note: Insufficient sleep = participants reported sleeping, on average, 7 hours or less. Sufficient sleep = participants reported sleeping, on average, 8 or 9 hours. Too much sleep = participants who reported sleeping, on average, 10 hours or more.
